# Platinum Concentration and Pathologic Response to Cisplatin-Based Neoadjuvant Chemotherapy in Muscle-Invasive Bladder Cancer

**DOI:** 10.1371/journal.pone.0155503

**Published:** 2016-05-17

**Authors:** Elizabeth A. Guancial, Deepak Kilari, Guang-Qian Xiao, Sohaib H. Abu-Farsakh, Andrea Baran, Edward M. Messing, Eric S. Kim

**Affiliations:** 1 Department of Medicine/James P. Wilmot Cancer Institute, University of Rochester, Rochester, New York, United States of America; 2 Department of Medicine, Medical College of Wisconsin, Milwaukee, Wisconsin, United States of America; 3 Department of Pathology, University of Rochester, Rochester, New York, United States of America; 4 Department of Biostatistics and Computational Biology, University of Rochester, Rochester, New York, United States of America; 5 Department of Urology, University of Rochester, Rochester, New York, United States of America; Johns Hopkins University, UNITED STATES

## Abstract

**Background:**

Platinum (Pt)-based chemotherapy is the standard of care for muscle-invasive bladder cancer (MIBC). However, resistance is a major limitation. Reduced intratumoral drug accumulation is an important mechanism of platinum resistance. Our group previously demonstrated a significant correlation between tissue Pt concentration and tumor response to Pt-based neoadjuvant chemotherapy (NAC) in lung cancer. We hypothesized that increased Pt concentration in radical cystectomy (RC) specimens would correlate with improved pathologic response to Pt-based NAC in MIBC.

**Methods:**

A cohort of 19 clinically annotated, archived, fresh frozen RC specimens from patients with MIBC treated with Pt-based NAC was identified [ypT0 (pathologic complete response, pCR), N = 4; ≤ypT1N0M0 (pathologic partial response, pPR), N = 6; ≥ypT2 (minimal pathologic response/progression), N = 9)]. RC specimens from 2 patients with MIBC who did not receive NAC and 1 treated with a non-Pt containing NAC regimen were used as negative controls. Total Pt concentration in normal adjacent urothelial tissue and bladder tumors from RC specimens was measured by flameless atomic absorption spectrophotometry.

**Results:**

Total Pt concentration in normal urothelium differed by tumor pathologic response (*P* = 0.011). Specimens with pCR had the highest Pt concentrations compared to those with pPR (*P* = 0.0095) or no response/progression (*P* = 0.020). There was no significant difference in Pt levels in normal urothelium and tumor between pPR and no response/progression groups (*P* = 0.37; *P* = 0.25, respectively). Conclusions: Our finding of increased intracellular Pt in RC specimens with pCR following NAC for MIBC compared to those with residual disease suggests that enhanced Pt accumulation may be an important determinant of Pt sensitivity. Factors that modulate intracellular Pt concentration, such as expression of Pt transporters, warrant further investigation as predictive biomarkers of response to Pt-based NAC in MIBC.

## Introduction

Muscle-invasive bladder cancer (MIBC) is a chemosensitive malignancy for which responses to cisplatin-based chemotherapy are seen in up to 50–60% of patients with advanced disease [1, 2]. However, the development of platinum (Pt) resistance is nearly universal. The historical average survival for advanced disease is 14 months with cytotoxic chemotherapy [3]. Clinical characteristics associated with poor prognosis have been identified in the setting of advanced bladder cancer [4]. Identification of predictive biomarkers of response to Pt-based therapy remains an active area of investigation. The cytotoxic effects of Pt agents are thought to be mediated by irreversible DNA damage. However, expression of DNA repair proteins, such as excision repair cross complementing 1 (ERCC1) and breast cancer susceptibility gene 1 (BRCA1), have not consistently been associated with response to Pt-based chemotherapy in MIBC [5–7]. Alternative approaches to predict sensitivity to Pt-based therapy involving multi-gene expression profiling with the coexpression extrapolation (COXEN) platform and panels of multiple immunohistochemistry (IHC)-detected protein biomarkers are currently being studied in MIBC treated with Pt-based NAC [8, 9].

Reduced intratumoral drug accumulation is an important *in vitro* mechanism of Pt resistance and has been demonstrated in cisplatin-resistant bladder cancer cell lines [10]. Previous work by our group demonstrated a significant correlation between tissue Pt concentration and tumor response to Pt-based NAC in lung cancer [11]. While Pt-based NAC is associated with an improvement in overall survival (OS) for MIBC, this treatment approach is underutilized in bladder cancer for multiple reasons. Prospective identification of bladder tumors most likely respond to Pt-based NAC would alter the risk to benefit ratio of chemotherapy and may significantly impact the clinical management of MIBC by increasing rates of NAC use in patients most likely to benefit. We hypothesized that increased Pt concentration in RC specimens would correlate with improved pathologic response to Pt-based NAC in MIBC. In this study, tissue Pt concentration was measured using flameless atomic absorption spectrophotometry (FAAS) in MIBC as well as normal urothelial tissue from RC specimens exposed to Pt-based NAC and correlated with pathologic response.

## Materials and Methods

### Patients and tissue specimens

A cohort of 19 clinically annotated RC specimens from patients with MIBC treated with Pt-based NAC was identified at the University of Rochester Medical Center (URMC) under an URMC Research Subjects Review Board (RSRB)-approved protocol (UMLT-13102). The URMC RSRB issued a waiver of consent for this protocol because subjects had previously signed written consent for their tissue to be banked in the URMC Urologic Tissue Bank (URMC RSRB-approved protocol #25352) for future translational research purposes; the protocol was categorized as minimal risk by the RSRB; data was analyzed in a de-identified manner; and the RSRB felt that the waiver would not adversely affect the rights and welfare of subjects, in accordance with the Declaration of Helsinki. Under protocol UMLT-13102, an additional RC specimen from a patient with MIBC who received a non-Pt containing NAC regimen and 2 RC specimens from patients with MIBC who did not receive NAC were analyzed as negative controls. Archived fresh frozen tissue was available for 19 cases. Non-tumor bearing adjacent bladder tissue (“normal” bladder urothelium) and either carcinoma or dysplasia, in the case of RC specimens that had a complete pathologic response following NAC (pCR; ypT0N0M0), were identified by a urologic pathologist. Pathologic partial response (pPR) indicated the presence of non-muscle invasive bladder cancer (NMIBC; ≤ypT1N0M0) in RC specimens. The presence of ≥ypT2 bladder cancer in the RC specimen was referred to as “minimal response/progression.”

### Tissue platinum measurement

Tumor tissue, when available, and non-malignant urothelial tissue adjacent to residual bladder tumors, or from where the tumor had previously been located in the case of pCR, was identified in RC specimens by a urologic pathologist. Tissue was weighed and digested in benzethonium hydroxide at 55°C to homogenize the sample. Total platinum content in each specimen was analyzed by FAAS as previously described [12]. Serial dilutions of a certified stock platinum standard (987 μg/mL; Sigma, St Louis, MO) were measured by FAAS to generate a linear standard curve. Platinum concentration was reported as absorbance unit per milligram of tissue for each specimen as previously described [11].

### Statistical analysis

The primary objective of this study was to determine if there was a relationship between Pt tissue concentration in MIBC and normal adjacent bladder urothelium from RC specimens and pathologic response following Pt-based NAC in MIBC. Kruskal-Wallis tests were used to compare the distributions of Pt concentrations by pathologic response (pCR, pPR, or “minimal response/progression”) and the number of cycles of NAC. Nonparametric Wilcoxon rank-sum tests were used to compare the distributions of Pt concentration in benign urothelium by gender and histology. Hypothesis tests were two-sided and conducted at the 0.05 level of significance. Spearman correlation coefficients were used to assess correlations in the measured Pt concentration between available matched normal and tumor specimens. SAS 9.4 (SAS Institute, Inc. Cary, NC) and GraphPad PRISM (version 6.05; GraphPad Software, La Jolla, CA) were used for analyses.

## Results

### Patient and tumor characteristics

Table 1 lists the characteristics of 19 evaluable patients with MIBC who underwent RC following Pt-based NAC. Median age of patients was 67 years; 63% were men and 37% were women. The majority of patients had clinical T2 (cT2) disease (85%); one patient had cT4, 1 patient had radiographic positive lymph nodes, and 1 patient had radiographic distant metastases, all before Pt-based chemotherapy. One patient (5%) received carboplatin-based NAC; the remainder (95%) received cisplatin-based NAC. Most patients (69%) treated with cisplatin received the combination of gemcitabine and cisplatin (GC). The numbers of cycles of NAC prior to RC ranged from 2 to 4, with the majority of patients (89%) receiving ≥3 cycles.

**Table 1:** Demographic and clinical characteristics of evaluable patients.

Characteristic	No. of Patients (N = 25)	%
Age, years		
Median	66	
Range	52–82	
Sex		
Male	17	68%
Female	8	32%
Ethnicity		
White	18	72%
African-American	1	4%
Unknown	6	24%
Clinical stage		
cT2N0M0	22	88%
cT4N0M0	1	4%
cTany N+ M0	1	4%
cTany Nany M+	1	4%
TURBT Histology		
UC	8	32%
High-grade papillary UC	11	44%
UC with squamous differentiation	3	12%
UC with micropapillary differentiation	1	4%
UC with mixed nested variant	1	4%
Small cell carcinoma	1	4%
Neoadjuvant chemotherapy		
Cisplatin-based		
GC	16	64%
MVAC	2	8%
GC and MVAC	1	4%
Cisplatin-etoposide	1	4%
Cisplatin-unknown	2	8%
Carboplatin-based		
G-carbo	2	8%
Carbo-unknown	1	4%
NAC cycles		
</ = 3	15	60%
> 3	8	32%
unknown	2	8%
Pathologic response to NAC at RC		
ypT0	5	20%
ypTis, ypTa, ypT1	8	32%
ypT2 or higher	12	

Abbreviations: GC, gemcitabine and cisplatin; G-carbo, gemcitabine and carboplatin; G-taxol, gemcitabine and paclitaxel; MVAC, methotrexate, vinblastine, adriamycin, and cisplatin; NAC, neoadjuvant chemotherapy; UC, urothelial carcinoma.

Among the 19 RC specimens, 4 had pCR, 6 had pPR, and 9 had minimal response/progression. High-grade papillary urothelial carcinoma (UC) was present in 48% of transurethral bladder tumor (TURBT) specimens and pure UC in 26%. Two cases had UC with squamous cell differentiation (11%), 1 with micropapillary differentiation (5%), and 1 with a mixed nested variant (5%). The histology present in the TURBT specimens of the 4 cases with pCR at the time of RC included 2 cases of high-grade papillary UC, 1 pure UC, and 1 small cell carcinoma of the bladder (Table 2).

**Table 2:** Clinical and pathologic variables and measured tissue platinum concentration stratified by pathologic stage.

Sex	Clinical stage	Histology	NAC agents	NAC cycles	Pathologic stage	Pt concentration (AU/mg): Benign urothelium	Pt concentration (AU/mg): Tumor
F	cT2N0M0	HG papillary UC	GC	2	ypT0N0	0.003329412	n/a
F	cT2N2M0	HG papillary UC	GC	4	ypT0N0	0.002541	n/a
M	cT2N0M0	UC	GC	3	ypT0N0	0.00835	n/a
M	cT2N0M0	small cell carcioma	cis-etoposide	3	ypT0N0	0.006623077	n/a
F	cT2N0M0	HG papillary UC	GC	3	ypTisN0	0.001942222	n/a
M	cT2N0M0	HG papillary UC, focal SqD	MVAC and GC	4	ypTisN0	0.001546667	n/a
F	cT4N0M0	HG papillary UC	MVAC	4	ypTaN0	0.000561538	0.000783871
M	cT2N0M0	HG papilary UC	GC	3	ypT1N0	0.00215625	0.001322727
M	cT2N0M0	HG papillary UC	G-carbo	3	ypTisN1	0.002039	n/a
F	cT2N0M0	UC	GC	3	ypTisN2	0.001169	n/a
F	cT2N0M0	UC	GC	2	ypT2aN0	0.001466667	0
M	cT2N0M0	HG papillary UC	GC	4	ypT2aN0	0.000836364	0.001658824
M	cT2N0M0	UC	GC	3	ypT3aN0	0.00213913	0
M	cT2N0M0	HG papillary UC	GC	4	ypT2bN1	0.00328511	n/a
M	cT2N0M0	UC	cis-?	3	ypT3N1	0.001234211	0.000260465
F	cT2N0M0	UC, mixed nested variant	GC	3	ypT3aN1	0.0025	n/a
M	cT2N0M0	HG papillary UC, focal SqD	GC	3	ypT3aN1	0.002963415	0.000641176
M	cT2N0M1	UC, focal micopapillary	MVAC	4	ypT4N1	0.002619	n/a
M	cT2N0M0	HG papillary UC	GC	4	ypT4N2	0.001093333	0

Abbreviations: AU/mg, absorbance units divided by milligram of tissue; carbo, carboplatin; cis, cisplatin; F, female; GC, gemcitabine, cisplatin; G-carbo, gemcitabine, carboplatin; HG, high grade; M, male; MVAC, methotrexate, vinblastine, adriamycin, cisplatin; n/a, not available; NAC, neoadjuvant chemotherapy; Pt, platinum; SqD, squamous differentiation; TURBT, transurethral resection of bladder tumor; UC, urothelial carcinoma.

### Tissue platinum concentration and tumor response

**Fig 1:**
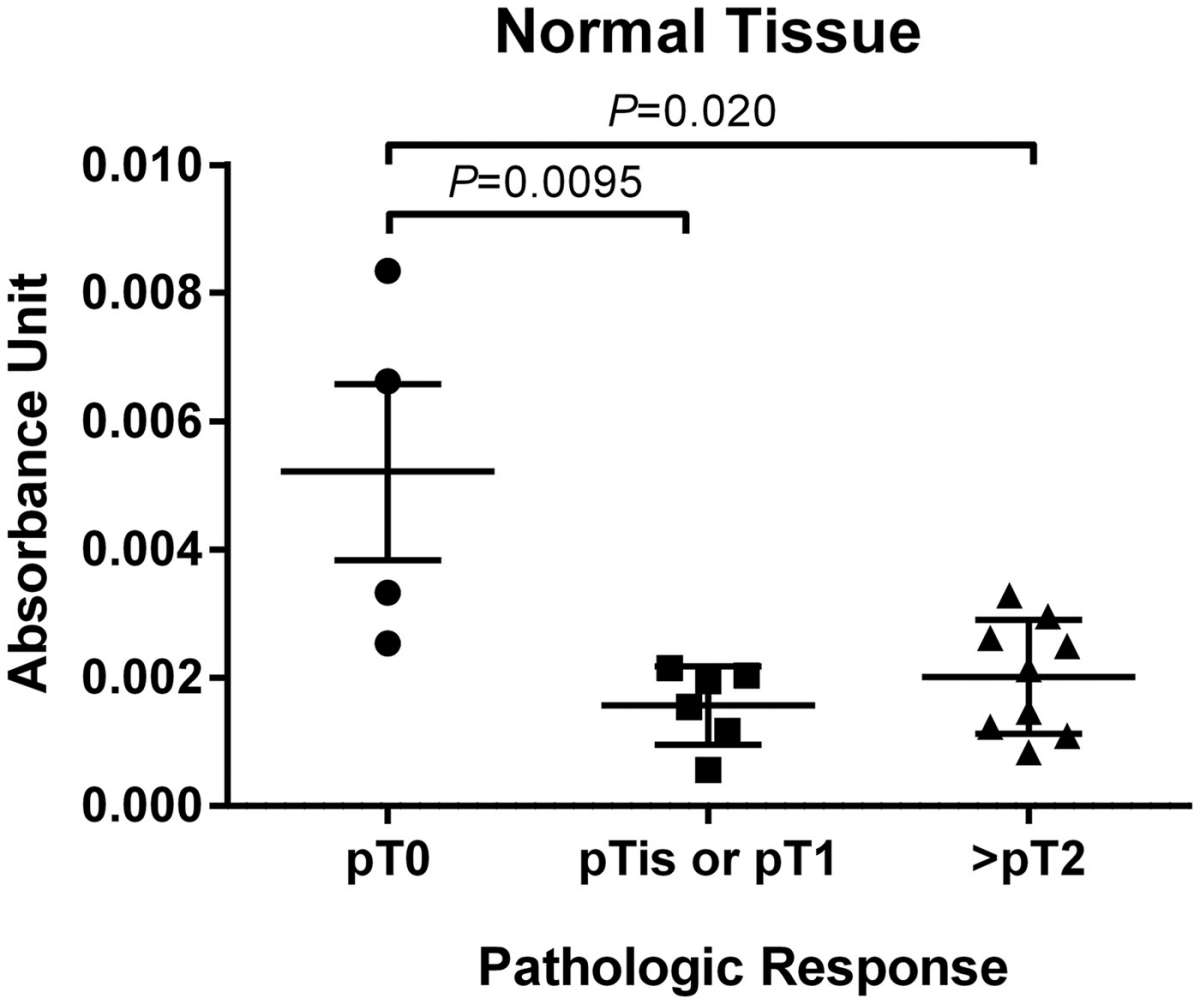
Total tissue Pt concentration reported in absorbance units per milligram of tissue in normal bladder tissue from RC specimens grouped by pathologic response. Bars in the plots show the mean (standard deviation).

**Fig 2:**
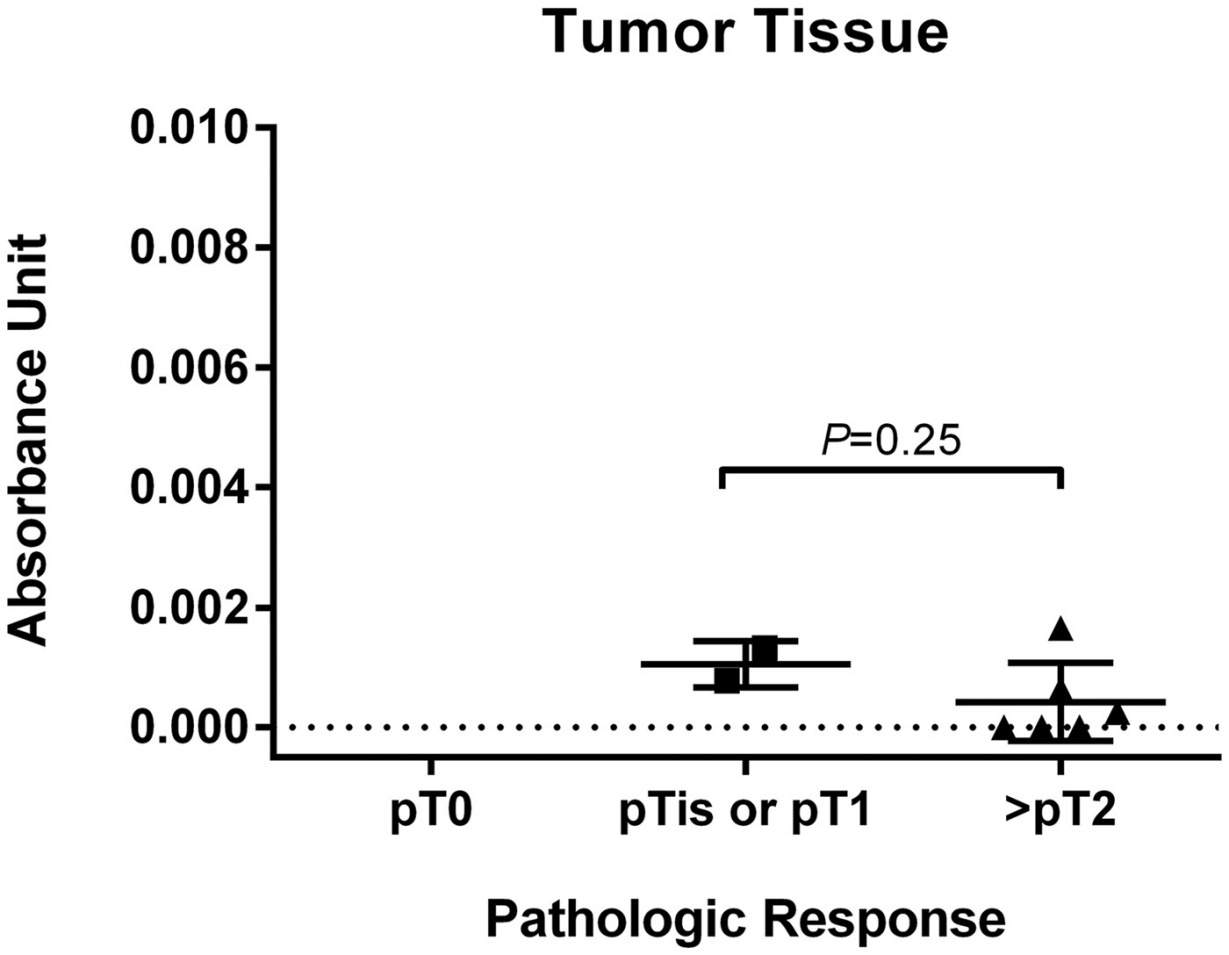
Total tissue Pt concentration in bladder tumors reported in absorbance units per milligram of tissue in tumor tissue from RC specimens grouped by pathologic response. Bars in the plots show the mean (standard deviation).

Absorbance units as measured by FAAS in the 3 negative control specimens not exposed to a Pt-agent prior to RC were similar to the background readings. Tissue Pt concentration was measured in 19 benign urothelial RC specimens and 8 paired RC tumor specimens treated with Pt-based NAC. Total Pt concentration in normal urothelial tissue significantly differed by tumor pathologic response (*P* = 0.011). Pt concentration in normal urothelial tissue from the 4 cases with pCR following NAC was significantly greater compared to Pt concentration in normal bladder tissue from cases with pPR (N = 6) (*P* = 0.0095) and cases with minimal response/progression (N = 9) (*P* = 0.020) (Fig 1). There was no significant difference in Pt levels in normal tissue between the pPR and no response/progression cohorts (*P* = 0.37). Similarly, Pt levels in tumor tissue from the pPR and no response/progression cohorts were not statistically different (*P* = 0.25) (Fig 2). No correlation was observed between the measured Pt concentration in available matched normal and tumor specimens (correlation = -0.17; *P* = 0.67). Neither gender, histology (pure UC versus high grade papillary UC), nor number of cycles of NAC (2, 3, or 4) were associated with Pt concentration in benign urothelial tissue (*P* = 0.47; *P* = 1.0; *P* = 0.49, respectively).

## Discussion

This proof-of-principal study demonstrates that Pt concentration measured by FAAS in benign adjacent urothelial tissue correlates with MIBC pathologic response following treatment with Pt-based NAC. Specifically, post-NAC cystectomy specimens with pCR had higher Pt concentrations in the surrounding normal urothelium than cases with any degree of residual MIBC or dysplasia. Importantly, while there was no correlation between Pt concentration in available paired benign and malignant tissues, cases with a complete pathologic response by definition had no residual tumor available for such testing. Our hypothesis was that increased intratumoral Pt concentration would result in the favorable pathologic regression of these tumors. However, an expected outcome of our experiemtns is that we were unable to test this hypothesis with the available tissue because no tumor remained from those cases that experienced the most dramatic response to chemotherapy.

The findings from our current study in MIBC support previous work performed by our group in non-small cell lung cancer (NSCLC), where FAAS-measured tissue Pt concentration correlated with percent reduction in tumor size based on pre- and post-NAC computed tomography scans [11]. Higher Pt concentration in NSCLC specimens was associated with prolonged time to recurrence (TTR), progression-free survival (PFS), and overall survival (OS) [11]. Variables such as specific Pt agent, tumor histology, number of cycles of chemotherapy, and length of time between last chemotherapy and surgical resection did not impact the correlation [11]. Due to the limited size of our current MIBC cohort and incomplete clinical information, including time from last chemotherapy to cystectomy and relapse or survival data, we were unable to assess the relationship between chemotherapy-related variables, pathologic response, and clinical outcomes such as TTR, PFS, and OS. However, absence of tumor in RC specimens compared with residual tumor is a known favorable prognostic factor associated with reduced risk of recurrence in patients with MIBC [13]. Thus, it is likely that patients in our cohort with pCR experienced improved disease-specific outcomes compared to those with residual carcinoma or dysplasia [14].

Similar to MIBC, Pt-doublet combination chemotherapy is the main systemic treatment for NSCLC. The use of Pt-based NAC is associated with an absolute improvement in OS of approximately 5% at 5 years for both diseases, though pCR rates with cisplatin-based NAC are lower in NSCLC compared with MIBC, 4% versus 29–49%, respectively [15–19]. Debate exists regarding the optimal use of NAC for MIBC, despite level 1 evidence of improved OS from randomized, phase III studies [20]. Among the multiple concerns is that toxicity from Pt-based regimens could jeopardize curative-intent RC or result in increased peri-operative morbidity, though this has not been borne out in randomized studies of RC with or without NAC [21][14].

A significant limitation to the use of NAC in MIBC and other Pt-sensitive malignancies is the lack of clinically useful predictive biomarkers of response to Pt-based chemotherapy. Unlike targeted therapy where the mechanism of action may be dependent upon a more limited set of molecular aberrations, such as HER2 overexpression and response to trastuzumab in breast and gastric cancers, cytotoxic effects from Pt agents are dependent upon a host of factors, including intravenous delivery of the agent to the tumor and the cellular response to DNA damage [22]. The DNA damage repair response involves coordination between multiple molecular pathways, such as nucleotide excision repair, mismatch repair and base excision repair, and it is generally thought that defective repair activity may be associated with enhanced cytotoxic effects from Pt agents. However, protein or mRNA expression of factors like excision repair cross-complementation group 1 (ERCC1) have not consistently been shown to correlate with response to Pt agents in NSCLC or BC [23–25]. Thus, alternative strategies are needed to identify predictive biomarkers for Pt agents.

Potential intracellular mechanisms associated with Pt resistance in BC could be broadly categorized as reduced influx of drug, increased efflux, and enhanced tolerance and/or repair of Pt-induced DNA damage [26]. The roles of Pt transporters, specifically copper related transporter 1 (CTR1), CTR2, ATP7A and ATP7B, are increasingly being recognized as important determinants of intracellular Pt concentration and potentially its cytotoxic effects. ATP7A and ATP7B are copper transporting ATPases implicated in Pt efflux [27]. A recent study demonstrated that loss of heterozygosity at the ATP7B locus was associated with an improved response to Pt-based chemotherapy in 17 patients with metastatic BC, suggesting that impaired efflux promoted cytotoxicity [28]. Landon et al. studied the role of CTR1 in mediating the previously demonstrated synergistic effects of hyperthermia and cisplatin in a BC cell line model [29], and found that the most cisplatin-sensitive BC cell lines had increased CTR1 expression. CTR1 wild type BC cell lines compared with CTR1-/-BC cell lines had more Pt accumulation. This difference was further amplified by hyperthermia where exposure to heat promoted CTR1 multimerization with and subsequent influx of cisplatin.

The relationship between CTR1 expression and tissue Pt concentration has been previously studied in NSCLC by our group [30]. We observed that CTR1 as measured by IHC was differentially expressed in 30 NSCLC tumors following Pt-based NAC. Both Pt concentration (*P* = 0.058) and tumor response (*P* = 0.016) were reduced in a subset of patients with undetectable tumor CTR1 expression compared to those with any level of CTR1 expression. Recent work by our group in a separate cohort of 47 patients with MIBC treated with Pt-based NAC demonstrated that increased CTR1 expression in both TURBT (*P* = 0.007) and RC (*P* = 0.02) specimens significantly correlated with pathologic responses [31]. The expression of CTR1 in matched TURBT and RC specimens was not significantly different (*P* = 0.55). Pt concentration was not measured in this study.

Our study design and conclusions were limited by our assumption that Pt concentration in normal urothelial tissue is a surrogate for Pt concentration in RC specimens with pCR. However, we acknowledge that this may be incorrect. While we did not observe a correlation between measured Pt in matched normal and tumor specimens or a difference in Pt concentration between specimens with partial response versus no response/progression of disease, this was a very small sample size that importantly lacked cases with pCR since we did not have access to serial RC specimens during the course of NAC. We were unable to assess the impact, if any, of different Pt agents due to the limited number of specimens that received carboplatin or length of time from last NAC to cystectomy due to limited clinical data for cases that received NAC outside of our institution but underwent RC at URMC. Unlike the NSCLC cohort previously studied by us [11], this population was fairly homogeneous with regards to histology (primarily urothelial carcinoma) and Pt agent (cisplatin) administered. Therefore, it is less likely that variability from these factors influenced our results.

In conclusion, increased Pt concentration was observed in urothelial tissue from RC specimens with pCR compared to those with residual disease following Pt-based NAC for MIBC. This finding suggests that enhanced Pt accumulation may be an important determinant of Pt sensitivity. Factors that modulate intracellular Pt concentration, such as expression of influx or efflux Pt transporters like CTR1, warrant further investigation as predictive biomarkers of response to Pt-based NAC in MIBC.
